# Invited Review: Antimicrobial Use and Antimicrobial Resistance in Pathogens Associated with Diarrhea and Pneumonia in Dairy Calves

**DOI:** 10.3390/ani12060771

**Published:** 2022-03-18

**Authors:** Xin Zhang, Xia Yi, Haohua Zhuang, Zhaoju Deng, Chong Ma

**Affiliations:** College of Veterinary Medicine, China Agricultural University, Beijing 100193, China; woshixiaoshouyi@gmail.com (X.Z.); yixia@cau.edu.cn (X.Y.); zthcau@163.com (H.Z.)

**Keywords:** dairy calf, antimicrobial use, antimicrobial resistance, calf diarrhea, calf pneumonia

## Abstract

**Simple Summary:**

Antimicrobial use (AMU) is the most important driver of antimicrobial resistance (AMR), and AMU in dairy calves accounts for a substantial amount of total AMU on dairy farms. However, an overview of AMU and AMR in dairy calves is lacking for the design of an AMU stewardship program. In this review, we summarize AMU and AMR data for dairy calves. We found large variation in AMU among herds in different regions, which indicates possibilities for reducing AMU. Antimicrobial resistance seemed to be associated with the types of antimicrobials used in specific regions. Farm type (conventional vs. organic) was associated with AMU but not with AMR. Management factors, such as feeding of calves and prophylactic use of antimicrobials, also related to AMR. This review provides an overview of AMU and AMR data for dairy calves and outlines opportunities for antimicrobial stewardship in dairy calves.

**Abstract:**

Antimicrobial use (AMU) is the major driver of antimicrobial resistance (AMR) among bacteria in dairy herds. There have been numerous studies on AMU and AMR in dairy cows; however, studies on AMU and AMR in dairy calves are limited. A comprehensive overview of the current state of knowledge of AMU and AMR among pathogens in dairy calves is important for the development of scientifically supported and applicable measures to curb antimicrobial use and the increasing risk of AMR. Therefore, we performed a systematic review of research on AMU and AMR in dairy calves. A total of 75 publications were included, of which 19 studies reported AMU data for dairy calves and 68 described AMR profiles of the four most prevalent bacteria that are associated with calf diarrhea and calf pneumonia. Large variation in AMU was found among herds across different regions. There seems to be a positive association between exposure to antimicrobials and occurrence of resistance. Most AMU was accounted for by treatment of diseases, while a small proportion of AMU was prophylactic. AMU was more common in treating calf diarrhea than in treating pneumonia, and the resistance rates in bacteria associated with diarrhea were higher than those in pathogens related to pneumonia. Organic farms used significantly fewer antimicrobials to treat calf disease; however, the antimicrobial resistance rates of bacteria associated with calf diarrhea and pneumonia on both types of farms were comparable. Feeding waste or pasteurized milk was associated with a higher risk of AMR in pathogens. Altogether, this review summarizes AMU and AMR data for dairy calves and suggests areas for future research, providing evidence for the design of antimicrobial use stewardship programs in dairy calf farming.

## 1. Introduction

Antimicrobial use is one of the main drivers of antimicrobial resistance in bacteria [1]. A number of studies have quantified antimicrobial usage (AMU) in dairy herds [2,3,4,5]. The largest amount of AMU in dairy herds was accounted for by mastitis and dry cow therapy, followed by treatment of calf diseases [2,5]. However, AMU in calves has scarcely been reported [5,6]. Redding et al. [5] found that AMU for treatment of calf diseases accounted for approximately 11.73% of the total AMU on dairy farms. It is therefore important to quantify AMU in dairy calves.

Calf diarrhea and pneumonia are the two primary diseases in dairy calves [7]. Sawant et al. [8] found that most of the AMU in dairy calves could be attributed to treatment of calf diarrhea (36% of the AMU for calves) and pneumonia (25% of the AMU for calves). There are several pathogens involved in calf diarrhea, of which *Escherichia coli* and *Salmonella* spp. are the two most prevalent pathogens associated with antimicrobial use [9,10,11]. Meanwhile, the most common bacterial pathogens for calf pneumonia are *Mannheimia haemolytica* and *Pasteurella multocida* [12,13]. These pathogens contributed to the majority of AMU in calves.

Antimicrobial resistance patterns among these pathogens in the context of calf diseases have been described in different regions [14,15,16]. In addition, proportionally higher levels of antimicrobial resistance in *Escherichia coli* and *Salmonella enterica* from dairy calves have been reported [17,18,19]. Antimicrobial resistance genes harbored in these pathogens could be a potential reservoir of AMR genes on dairy farms [14]. 

In order to reduce antimicrobial (ab)use and the antimicrobial resistance of pathogens related to dairy calves, it is vital to investigate current antimicrobial use and the antimicrobial resistance of pathogens in dairy calves. In this review, we summarize the data available for aspects of antimicrobial usage and the antimicrobial resistance profiles of bacteria associated with calf diarrhea and pneumonia.

## 2. Materials and Methods

### 2.1. Literature Search

A systematic literature search was performed in three public databases (PubMed, Scopus and Web of Science) according to “PRISMA 2020 explanation and elaboration: updated guidance and exemplars for reporting systematic reviews” [20]. Search terms reflecting antimicrobial use and antimicrobial resistance among pathogens in dairy calves were combined with the AND operator in a search constrained on title and abstract: (dairy calf OR dairy calves) AND ((antimicrobial use OR antibiotic use OR drug use) OR (antimicrobial resistance OR antibiotic resistance OR drug resistance)). The search was limited to publications written in English and published from 1 January 1990 to 26 August 2021. 

### 2.2. Study Selection

Studies retrieved from the three public databases were imported into Rayyan [21] for screening of eligible studies by two researchers independently. Duplicates were removed prior to the screening of eligible publications. Discrepancies were discussed during group meetings and a consensus was reached among all the co-authors. The selection of studies was made according to the following criteria:

1. Studies should primarily focus on antimicrobial usage in dairy calves or antimicrobial resistance of pathogens associated with calf diarrhea and calf pneumonia;
2. Quantitative data on AMU or AMR for at least one class of antimicrobials should be provided;
3. Studies should be written in English;
4. Studies should consist of original research;
5. Full texts should be available;
6. Statistical analysis was appropriate for the study design.

**Figure 1:**
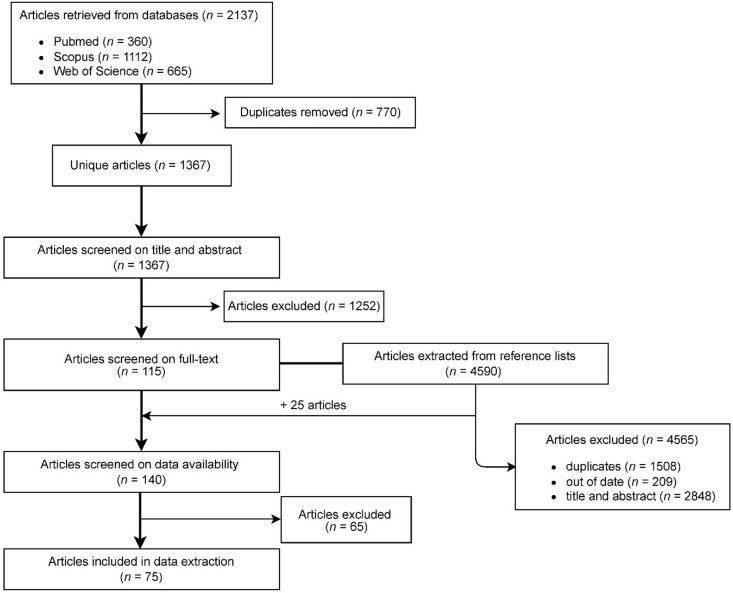
Flow diagram of the selection process for studies (*n* = number of studies).

The first two authors performed the data extraction and an extra check for accuracy and completeness was made by the corresponding authors. Due to the high heterogeneity in terms of study design, definitions and data calculations in the publications included, we performed a review instead of a meta-analysis and summarized the quantitative data wherever possible. Discrepancies regarding the process of study selection, risk of bias assessment and data extraction were resolved in group discussions with all the co-authors until consensus was reached. Meanwhile, the references of the publications that remained when it came to screening full texts were also scanned in the same procedure (Figure 1) in search of eligible studies. 

### 2.3. Data Extraction

Data were extracted from texts, tables, and figures using WebPlotDigitizer (https://automeris.io/WebPlotDigitizer/, accessed on 15 October 2021) wherever needed. For this review, we specifically focused on calf diarrhea and calf pneumonia since those are the two most prevalent diseases in dairy calves. We summarized the antimicrobial usage information in all the studies with AMU data.

## 3. Results

### 3.1. Literature Search

A total of 2137 studies were retrieved from PubMed (360), Scopus (1120) and Web of Science (665). After screening for the title and abstract, there were 115 full text papers included for the screening of full texts. The references in these 115 publications were also screened for eligible studies and an additional 25 studies from the references were included in the data availability assessment. A total of 75 publications were finally included in the data extraction. A flow diagram for the study selection process is presented in Figure 1. There were 19 papers primarily reporting AMU in dairy calves, 68 papers on the antimicrobial resistance of pathogens isolated from dairy calves and 12 papers touched on both topics. A total of 39 papers were cross-sectional studies, 26 were cohort studies, 2 studies were case–control studies and 1 study was a randomized controlled trial. The majority of the papers on antimicrobial resistance were concerned with calf diarrhea (65 studies) and three papers were concerned with pneumonia. The pathogens involved in calf diarrhea were *E. coli* (48 studies), *Samonella* (12 studies), *Campylobacter* (4 studies) and *Clostridium perfringens* (1 study) as well as *Enterobacter* spp. (1 study), while *Pasteurella* spp. (3 studies) and *Mannheimia* spp. (1 study) were the most frequently reported pathogens in calf pneumonia based on these 4 studies. Antimicrobial resistance was tested using the broth microdilution method (31 studies) or the disk diffusion method (28 studies). Resistance against seven classes of antimicrobials (*β*-lactam, sulfonamides, aminoglycosides, phenicols, tetracyclines, quinolone and macrolides) was found in these pathogens. There were six studies that detected antimicrobial resistance genes in bacterial pathogens; these resistance genes included blaCMY, blaCTX-M, blaTEM, tetA, tetB, tetM, tetO, strA, strB, aadA, sul1, sul2, cat, floR, cfr and ermB.

### 3.2. Antimicrobial Use

#### 3.2.1. Antimicrobial Use at Herd Level

There were six studies that quantified AMU at herd level [8,22,23,24,25,26]; on average, antimicrobials would be used to treat calves with diseases in 40.65% (*n* = 2609; 2.5–97.5% quantile: 21.73–71.15%) herds. About 50% (*n* = 1328; 2.5–97.5%: 45.48–50.75%) of the farms would use antimicrobials to treat calves with respiratory symptoms, while only 36.77% (*n* = 1009; 2.5–97.5%: 21.70–74.51%) farms would use them to treat calves with digestive disorders. Organic farms (2 studies, 405 farms) were statistically less likely to adopt antimicrobial therapy as compared with conventional farms (2 studies, 2330 farms; 25.00% vs. 35.62%), especially in cases of calf diarrhea (1 study, 32 farms vs. 99 farms; 21.88% vs. 78.79%) [22]. An estimated 17.75% (1 study, 408 farms; 2.5–97.5%: 16.08–19.00%) of calves on conventional farms (*n* = 2298) received antimicrobial treatment [27], while 13.00% (1 study, 40 farms; 2.5–97.5%: 16.08–19.00%) of calves on organic farms (*n* = 306) received treatment [22]. 

Overall, the most frequently used antimicrobials for dairy calves were sulfachlorpyridazine, ampicillin and enrofloxacin [28], while the most commonly used antimicrobials for treatment of diarrhea were sulfamethazine and oxytetracycline and for the treatment of pneumonia the most commonly used were florfenicol and tilmicosin [26]. The antimicrobials used to treat calf diarrhea and calf pneumonia are listed in Table 1.

#### 3.2.2. Antimicrobial Use at Calf Level

There were 8 publications describing AMU at the calf level. About 35.50% (*n* = 5233) calves received prophylactic antimicrobials, while 35.66% of the calves with diseases were treated with antimicrobials. Most AMU was attributed to treatment of diseases, while prophylactic AMU was relatively low.

About 29.41% (*n* = 702) of calves with calf diarrhea were treated with antimicrobials (number of studies: 4) and 60.77% (*n* = 361) of calves with pneumonia were also treated (number of studies: 3) (Table 2). Jarrige et al. [25] found antimicrobials in 35.50% (*n* = 370) of milk samples from milk fed to calves. About 35.81% (*n* = 433) of the calves received antimicrobial treatment at least once. Treatment of calf diarrhea (317/669, 47.38%) and pneumonia (221/669, 33.03%) were the major reasons for antimicrobial use [27]. Most calves received antimicrobials, and the majority of calves received two to four treatments; young calves received antimicrobial treatment more frequently than older calves [28]. Detailed data are summarized in Table 2.

#### 3.2.3. Antimicrobial Use

A total of 19 studies described antimicrobial usage data for dairy calves; however, only 5 studies provided detailed data suitable for statistical analysis. The AMU data were recorded for each antimicrobial or class of antimicrobials in the five studies included in this review, therefore we summarized AMU for classes of antimicrobials (or for each antimicrobial wherever possible). The AMU data in this review were based on veterinary treatment records and questionnaire data collected by researchers. Meanwhile, methods for calculating AMU across regions were different; we took used daily doses (UDD) as the unit of AMU for this review wherever applicable; other units of AMU were transformed to UDD accordingly. Total AMU for calf use was 0.19 animal-defined daily doses (mean ADDD) in the Netherlands [2], 0.30 (mean ADDD; median ADDD: 0.17) in Denmark [27] and approximately 9.74 (mean ADDD) in Pennsylvania [5]. 

The most commonly used classes of antimicrobials were sulfonamides (UDD: 32.71), amoxicillin–clavulanic acid (UDD: 16.00), sulfadimidine (UDD: 15.77) and florfenicol (UDD: 14.13). There were no records of lincomycin or lincosamides used to treat calves. The mean percentage of each antimicrobial used in dairy calves was calculated as the arithmetic mean of the percentages of each antimicrobial used for different purposes in all studies. Treating diseased calves was the major driver of AMU (90.5% of the farms, 1 study, 169 farms), while antimicrobials were also used for prevention of diseases on a small proportion of farms (11% of the farms) [34]. Detailed AMU data are summarized in Table 3 and Supplementary Table S1.

#### 3.2.4. Antimicrobial Resistance of Calf Diarrhea-Associated Pathogens

The antimicrobial resistance data for pathogens associated with calf diarrhea and pneumonia are summarized in the following section. There were various resistance patterns found in these studies. Given different definitions of multi-drug resistance patterns across studies, we only present the results for antimicrobial resistance to individual antimicrobials in this review.

According to the recommendations of first-line and second-line medication for treatment of calf diarrhea and pneumonia, antimicrobials belonging to seven different classes of antimicrobials, including *β*-lactam, sulfonamides, aminoglycosides, phenicols, tetracyclines, quinolones and macrolides, were selected for the summary of antimicrobial resistance. Most of the farms used antimicrobials belonging to *β*-lactam, sulfonamide and aminoglycoside, while reports on the remaining four classes of antimicrobials were scarce. The median proportion of antimicrobial-resistant isolates for all the four different species of bacteria was 0.26 (2.5–97.5% quantile: 0.10–0.99).

##### Calf Diarrhea

*E. coli*

Resistance to seven classes of antimicrobials was found. In general, high rates of resistance to cefalexin (100%; 2.5–97.5% quantile: 100–100%; 20 isolates), erythromycin (99%; 2.5–97.5% quantile: 99–99%; 176 isolates), amoxicillin (79%; 2.5–97.5% quantile: 30–100%; 687 isolates) and tetracycline (55%; 2.5–97.5% quantile: 16–96%; 10,335 isolates) were found, while resistance rates to the class of quinolone antimicrobials were relatively low (ranging from 9 to 15%). The numbers of isolates included in the studies resistant to cephalexin and erythromycin were limited.

*Salmonella* spp.

The number of studies on antimicrobial resistance to *Salmonella* spp. was lower than the number of studies on *E. coli*. High rates of resistance to oxytetracycline (89%; 2.5–97.5% quantile: 88–95%; 127 isolates), sulfamethoxazole (83%; 2.5–97.5% quantile: 1–96%; 653 isolates) and florfenicol (0.81; 2.5–97.5% quantile: 61–86%; 109 isolates) were found. No isolates were found that were resistant to amoxycillin or cefalexin among *Salmonella* spp. The resistance rates for most of the antimicrobials in *Salmonella* spp. were higher than those in *E. coli*.

Antimicrobial resistance data for each pathogen isolated from calf diarrhea and calf pneumonia are summarized in Table 4.

##### Antimicrobial Resistance of Bacteria Associated with Calf Pneumonia

There were only two studies that reported the antimicrobial resistance profiles of pathogens associated with calf pneumonia, with a limited number of bacterial isolates. *Pasteurella* spp. isolates were resistant only to colistin (24.7%, 74/301), erythromycin (5.5%, 16/301) and streptomycin (77.7%, 233/301) [16]. Catry et al. [15] investigated the distribution of *Pasteurella* spp. and *Mannheimia* spp. in the upper respiratory tracts of calves that were not treated with antimicrobials. This study only performed tests of antimicrobial resistance to tetracycline for these two pathogens. The detailed descriptive statistics of antimicrobial resistance data for *Pasteurella* spp. and *Mannheimia* spp. are summarized in Supplementary Table S2.

## 4. Discussion

Antimicrobial use in dairy calves is an important source of AMU in dairy cows. To illustrate the current status of AMU in dairy calves and AMR in pathogens associated with calf diarrhea and pneumonia, we summarized published data on AMU and AMR in pathogens related to these two diseases in calves.

We searched studies published in the years 1990–2021, which constitutes a long time span given that the dairy industry has evolved largely during these three decades. Therefore, results generated by comparisons of data collected in different regions over large time intervals should be interpreted with caution. Results obtained using different methods (AMR results from different methods), on the basis of a limited number of studies and from a limited number of farms should also be interpreted with care. 

Various methods for calculating AMU were used in the studies included in this review, which makes comparisons difficult. Each AMU calculation method has its merits; however, the diversity of methods made the comparison of antimicrobial use across regions difficult; therefore, standardized methods of calculation for AMU are needed. Schrag et al. [88] described approaches to standardize the calculation of AMU in dairy herds. Merle et al. [89] compared two different approaches for calculation of AMU and found inconsistent results for different antimicrobials using these two methods (UDDs were higher than ADDDs in most cases). Hyde et al. [90] found that AMU rates calculated as defined daily dose were generally higher than those calculated as defined course dose. Meanwhile, different definitions of multi-drug-resistance patterns were also found. Awosile et al. [84] and Pereira et al. [53] defined multi-drug resistance as being resistant to ≥3 different antimicrobials, while Sjöström et al. [36] defined multi-drug resistance as resistance to >3 different antimicrobials. These subtle differences in definitions made comparisons difficult. Unified standardized definitions of these terms should be made in order to communicate findings from different regions.

We found large variation in AMU among herds across regions, which indicates the possibility to further reduce AMU [6]. Total AMU for calves differs substantially across regions, being 0.19 animal-defined daily doses (mean ADDD) in the Netherlands [2], 0.17 (median ADDD) in Denmark [27] and approximately 9.74 (mean ADDD) in Pennsylvania [5]. Despite regulations in antimicrobial use in different regions, attitudes of farmers and veterinarians towards animal health and antimicrobial use also influence AMU [91,92]. According to the European regulation for organic dairy herds (Council Regulation (EC) No 834/2007), AMU is restricted to a maximum number of three treatments per cow per year. Currently, the number of treatments per calf was slightly higher than this standard [36]. AMU for calves in the US was higher by orders of magnitude than in Denmark and the Netherlands. One of the explanations for this could be that the EU imposes much stricter regulations on antimicrobial use than the US. Measurements to further reduce AMU in calves are needed. The first month is the period with the highest risk for AMU in calves [6]. As the treatment of diseases is the major driver of AMU in calves, successful prevention programs for calf diarrhea and pneumonia in the first month would largely reduce AMU in calves. In addition, the threshold of severity of the diseases upon the initialization of adopting antimicrobial therapy also contributes to AMU [88]. Meanwhile, herd size is a risk factor for treatment incidence in conventional herds [27]. With increasing herd sizes worldwide, it seems inevitable to take this into consideration in attempting to strike a balance between calf health and AMU stewardship.

Exposure to antimicrobials increases the risk of AMR. Resistance rates for the most commonly found antimicrobials were generally higher than those for rarely used antimicrobials. Antimicrobial residues in calf feedings could be a source of AMR genes from dams. There was large variation in the use of antimicrobials as additives in milk used to feed calves [8,93]; 165 out of 655 farms (25.17%) would add antimicrobials to milk replacer [26]. Feeding milk with antimicrobial residues (waste milk, pasteurized milk) to calves increases the rate of AMR [94,95,96,97]. Extended-spectrum *β*-lactamase-producing Enterobacteriaceae (92.9% of which were *E. coli*) were found in newborn dairy calves [33], which indicates the transmission of AMR genes from dams to calves. However, the transmission routes of AMR genes are not fully clear yet. Future research to quantify the contributions of dams, environmental sources and calves within the same pen would be useful in controlling the transmission of AMR genes in calves. Alternatives to antimicrobials, such as herbs, minerals and vitamins, might reduce AMR in the treatment of calf diseases [34].

Organic farms used fewer antimicrobials as compared with conventional farms, while AMR patterns of bacteria on both types of farms were similar [36]. One plausible explanation for this could be the transmission of AMR genes from milk delivered by dams harboring those AMR genes. Meanwhile, previous studies have shown that AMR is positively associated with AMU; therefore, control of AMR requires efforts in multifactorial aspects in addition to prudent use of antimicrobials.

The data on pathogens associated with calf pneumonia were rather limited; higher AMU was found in the treatment of calf pneumonia than in that of calf diarrhea, largely due to the prevalence of these diseases [27,89]. Therefore, future studies including data on calf pneumonia in large numbers of herds are needed in order to develop better tailor-made AMU stewardship programs.

AMU stewardship programs consist not only of animal health and AMR components; the costs of such programs for farms, customers and society, both short- and long- term, are also relevant. However, there is only limited research on this aspect. With respect to the prohibition of AMU, Lhermie et al. [98] found slightly increased costs for both farms and costumers.

## 5. Conclusions

The aim of this systematic review was to provide evidence that can be used to develop antimicrobial use stewardship programs. The heterogeneity in measurements and calculations of antimicrobial usage data made it challenging to perform a meta-analysis. There is large variation in antimicrobial use for calves among farms and antimicrobial resistance seems to be associated with the use of antimicrobials in calves. Future studies focusing on factors associated with antimicrobial use in calves and elucidation of the modes of transmission of antimicrobial resistance genes are needed in order to curb antimicrobial use and antimicrobial resistance.

## Figures and Tables

**Table 1:** Antimicrobial use for different purposes among different farm types. Number of farms with treatment divided by the total number of farms in brackets.

Purpose of Use	Farm Type	Percentage of Farms with Treatment	Country (Ref.)
Diarrhea	Conventional herd	78.79% (78/99)	US [22]
Diarrhea	Organic herd	21.88% (7/32)	US [22]
Gastro-intestinal	Conventional herd	35.99% (827/2298)	US [29]
Gastro-intestinal	Organic herd	31.05% (95/306)	US [29]
Pneumonia	Conventional herd	45.45% (15/33)	US [8]
Respiratory	Conventional herd	51.00% (1172/2298)	US [29]
Respiratory	Organic herd	46.08% (141/306)	US [29]
Treatment	Conventional herd	25.00% (1/3)	US [24]
Treatment	Conventional herd	40.00% (2/5)	France [25]
Milk replacer	Conventional herd	25.19% (165/655)	US [26]
Feed	Conventional herd	27.30% (104/381)	US [26]
Total		64.21% (6882/10,718)	

**Table 2:** Antimicrobial use for different purposes at calf level. Number of calves with treatment divided by the total number of calves on farms in brackets. These data are all from conventional herds.

Purpose of Use	Type of Calves	Percentage of Calves with Treatment	Country (Ref.)
Diarrhea	Calves (<90 days)	18.26% (310/1698)	Canada [30]
Diarrhea	Calves	36.15% (141/390)	US [8]
Diarrhea	Calves	84.20% (190/226)	US [26]
Diarrhea	Pre-weaned heifer calves	83.00% (61/73)	US [31]
Pneumonia	Calves	25.38% (99/390)	US [8]
Pneumonia	Calves	97.00% (198/204)	US [26]
Pneumonia	Pre-weaned heifer calves, 0–90 days	100.00% (64/64)	US [31]
Prevention	Calves	54.02% (47/87)	US [31]
First treatment	Calves in individual pens, feeding calves with milk or milk replacer	38.41% (63/164)	US [23]
First treatment	Calves in group pens, feeding calves with acidified milk ad libitum	17.46% (55/315)	US [23]
Second treatment	Calves in individual pens, feeding calves with milk or milk replacer	18.90% (31/164)	US [23]
Second treatment	Calves in group pens, feeding calves with acidified milk ad libitum	6.03% (19/315)	US [23]
Third treatment	Calves in individual pens, feeding calves with milk or milk replacer	8.54% (14/164)	US [23]
Third treatment	Calves in group pens, feeding calves with acidified milk ad libitum	0.95% (3/315)	US [23]
Treatment	Calves (from birth to weaning)	99.40% (4275/4301)	US [28]
Treatment	Calves	87.34% (69/79)	US [31]
Treatment	4.6 (2.0; 1 to 8) *	14.86% (11/74)	US [32]
Treatment	18.6 (2.0; 15 to 22) *	27.03% (20/74)	US [32]
Treatment	32.6 (2.0; 29 to 36) *	29.73% (22/74)	US [32]
Treatment	Calves (0–14 days) *	11.67% (21/180)	Germany [33]
Treatment with one type	Calves (from birth to weaning)	12.41% (534/4301)	US [28]
Treatment with two or more types	Calves (from birth to weaning)	88.15% (3793/4301)	US [28]
Milk replacer	Calves	56.32% (49/87)	US [31]
Total (diarrhea)		29.41% (702/2387)	
Total (pneumonia)		60.77% (361/594)	
Total (disease)		35.66% (1063/2981)	
Total		35.50% (5233/14,742)	

* Age/d (SD, min to max).

**Table 3:** Percentages of antimicrobials used for different purposes in dairy calves. Means were calculated as the arithmetic means of these percentages. There were three studies from the US and one study from France.

	Use Category	Treatment (US) [28]	Diarrhea (US) [26]	Pneumonia (US) [26]	Prevention (US) [26]	Treatment (US) [26]	Treatment(US) [23]	Treatment (US) [23]	Treatment (US) [23]	Treatment (US) [23]	Treatment (US) [23]	Treatment (US) [23]	Treatment (France) [25]	Mean
*β*-Lactam	*β*-Lactam						79.37%	12.73%	35.48%		7.14%			33.68%
	Amoxicillin				2.20%	28.60%								15.40%
	Amoxicillin/ Clavulanic acid												4.00%	4.00%
	Ampicillin	75.00%			0.50%	26.50%		12.73%						28.68%
	Penicillin				6.30%	89.90%	17.46%		6.45%				8.00%	25.62%
	Ceftiofur	3.30%	14%	18%	8.60%	87.80%	61.90%		29.03%		7.14%			28.72%
Macrolides	Macrolides						11.11%	23.64%	58.06%	52.63%	42.86%		1.00%	31.55%
	Erythromycin				1.10%	12.40%								6.75%
	Tilmicosin			20%	2.20%	38.50%								20.23%
	Tulathromycin	11.80%					11.11%	23.64%	54.84%	52.63%	35.71%			31.62%
	Tylosin				2.20%	27.30%			3.23%		7.14%			9.97%
Aminoglycosides	Aminoglycosides												11.00%	11.00%
	Gentamycin				80.00%	16.60%								48.30%
Tetracyclines	Tetracyclines						9.52%					33.33%	1.00%	14.62%
	Chlortetracycline/Sulfamethazine				25.80%	23.90%								24.85%
	Neomycin/ Oxytetracycline				7.60%	18.90%								13.25%
	Oxytetracycline		18%		8.00%	69.80%	9.52%					33.33%		27.73%
Phenicols	Phenicols						9.52%					33.33%		21.43%
	Florfenicol	15.60%		27%	2.50%	41.20%	9.52%					33.33%	1.00%	18.59%
Quinolones	Fluoroquinolone						3.17%	49.09%	6.45%	47.37%	21.43%	66.67%		32.36%
	Enrofloxacin	55.00%					3.17%	49.09%	6.45%	47.37%	21.43%	66.67%		35.60%
Sulfonamides	Sulfonamides				2.20%	55.00%		1.82%						19.67%
	Sulfonamides/ Trimethoprim												3.00%	3.00%
	Sulfachlorpyridazine	97.80%			0.30%	10.20%								36.10%
	Sulfamethazine		28%					1.82%						14.91%
Colistin	Colistin												6.00%	6.00%

**Table 4:** Antimicrobial resistance patterns for *E. coli* and *Salmonella* spp. isolated from dairy calves with diarrhea. The total number of studies and the corresponding references were listed for each pathogen. Number of resistant isolates divided by the total number of isolates for each antimicrobial are displayed. Critically important antimicrobials (according to WHO) with the highest priority are marked in bold and italic.

Class of Antimicrobial	Antimicrobial	*E. coli*		*Salmonella* spp.	
		Ref.	Proportion of Resistant Isolates	Ref.	Proportion of Resistant Isolates
***β***-lactam	Amoxycillin	[32,35,36,37,38,39,40]	0.79 (687/869; 0.30–1.00)	[28,41]	0.00 (0/37; 0.00–0.00)
	Amoxicillin/Clavulanic acid	[19,36,38,42,43,44,45,46,47,48]	0.24 (538/2228; 0.00–0.91)	[19,42,49,50]	0.26 (731/2769; 0.02–0.93)
	Ampicillin	[16,19,28,35,36,37,38,41,42,43,44,45,46,49,50,51,52,53,54,55,56,57,58,59,60,61,62]	0.21 (4281/19,993; 0.00–0.98)	[17,19,41,42,48,49,50,51,52]	0.75 (786/1046; 0.14–0.92)
	Cefalexin	[43]	1.00 (20/20; 1.00–1.00)	[41]	0.00 (0/19; 0.00–0.00)
	***Ceftiofur***	[19,35,36,37,41,42,49,50,51,52,53,54,55,56,57]	0.32 (2379/7457; 0.00–1.00)	[19,42,47,49,50,51]	0.75 (726/974; 0.03–0.98)
	***Ceftriaxone***	[19,42,49,50,51,52]	0.13 (467/3555; 0.05–0.81)	[19,42,49,50,60]	0.36 (1125/3121; 0.00–0.47)
	Cefalothin	[25,43,45,56,57,63,64]	0.11 (178/1596; 0.00–0.90)		
	Cefoxitin	[19,42,50,51,52,53]	0.29 (872/3027; 0.00–0.75)	[19,42,49,50,60]	0.40 (1194/3022; 0.09–0.49)
Sulfonamides	Sulfamethoxazole	[17,28,32,45,63,65,66,67]	0.12 (978/8123; 0.02–0.69)	[50,61,68]	0.83 (653/788; 0.01–0.96)
	Sulfisoxazole	[19,42,49,50,51,52]	0.39 (3999/10,133; 0.04–0.93)	[19,42]	0.50 (1131/2252; 0.51–0.77)
	Trimethoprim/Sulfamethoxazole	[19,28,35,36,37,38,41,42,43,49,50,51,52,53,54,55,56,57,58,59,60]	0.29 (2590/8832; 0.03–0.82)	[17,41,48,49,50,51,59]	0.23 (691/2992; 0.00–0.79)
Aminoglycosides	Amikacin	[19,42,49,50,51,52]	0.22 (793/3601; 0.01–0.87)	[69,70,71]	0.12 (92/783; 0.01–0.31)
	Gentamicin	[16,19,28,35,36,37,38,41,42,43,44,45,46,49,50,51,52,53,54,55,56,57,58,59,60,61,62]	0.16 (1814/11,473; 0.00–0.87)	[17,41,48,49,50,51,53,59]	0.29 (871/2959; 0.11–0.83)
	Kanamycin	[42,50,51,52,53,54,72,73,74]	0.37 (813/2183; 0.06–0.91)	[17,41,48,49,50,51,75]	0.76 (665/880; 0.05–0.92)
	Streptomycin	[17,28,34,35,38,40,43,44,45,47,48,49,50,51,52,53,54,57,58,59,60,61,62]	0.36 (7438/20,875; 0.04–0.91)	[19,42,49,50,51]	0.79 (681/861; 0.22–0.97)
	Neomycin	[29,42,50,51,73,76,77]	0.38 (816/2158; 0.09–0.94)	[19,42,49]	0.56 (99/178; 0.09–0.83)
Phenicols	Chloramphenicol	[19,28,35,36,37,41,42,49,50,51,52,53,54,55,56,57,58,59,60]	0.16 (2012/12,803; 0.03–0.85)	[19,42,47,49,50,51]	0.24 (650/2737; 0.01–0.94)
	Florfenicol	[19,35,36,41,42,49,50,51,52,53,54,55,74]	0.20 (874/4430; 0.00–0.98)	[78,79,80]	0.81 (109/135; 0.61–0.86)
Tetracyclines	Tetracycline	[19,28,35,36,37,38,41,42,43,44,45,49,50,51,52,53,54,55,56,57,58,59,60,61]	0.55 (10,335/18,669; 0.16–0.96)	[19,42,48,49,50,51,52,53]	0.55 (2766/5046; 0.21–0.99)
	Oxytetracycline	[16,35,40,43,72,77,81]	0.27 (386/1430; 0.04–0.89)	[82,83]	0.89 (127/143; 0.88–0.95)
Quinolone	***Ciprofloxacin***	[19,35,36,41,42,49,50,51,52,53,54,55]	0.09 (486/5212; 0.00–0.36)	[19,42,49,50,84]	0.17 (467/2805; 0.00–0.63)
	***Enrofloxacin***	[19,35,36,41,42,49,50,51,52,53,54,55]	0.15 (295/2013; 0.00–0.84)	[19,42]	0.08 (12/143; 0.00–0.09)
	***Nalidixic acid***	[19,35,36,37,41,42,49,50,51,52,53,54,55,56]	0.11 (694/6135; 0.00–0.76)	[15,23,33,73,85,86,87]	0.03 (78/2806; 0.00–0.05)
Macrolides	***Erythromycin***	[19]	0.99 (176/178; 0.99–0.99)		

## Data Availability

Data are contained within the article or in the Supplementary Materials.

## Supplementary Materials

The following supporting information can be downloaded at: https://www.mdpi.com/article/10.3390/ani12060771/s1, Supplementary Table S1: Quantity of each antimicrobial that was used for dairy calves. Antimicrobial usage were expressed in used daily doses (UDD). The mean of the AMU for each antimicrobial was the simple arithmetic mean of the AMU for each antimicrobial (2.5–97.5% quantile in brackets). There were 1 study in Canada, 2 studies in Ireland and 2 studies in Sweden. Supplementary Table S2: Antimicrobial resistance of bacteria associated with calf pneumonia. The number of resistant isolates divided by the total number of isolates and the corresponding 2.5–97.5% quantile were provided in brackets. Study on *Pasteruella* spp. was in Spain and *Mannheimia* spp. in Belgium, respectively. Critically important antimicrobials (according to WHO) with the highest priority were in bold and italic [6,15,16,30,36,99].
