# Supplementary material for: Invited Review: Antimicrobial Use and Antimicrobial Resistance in Pathogens Associated with Diarrhea and Pneumonia in Dairy Calves
Source: Animals (Basel). 2022 Mar 18;12(6):771. doi: 10.3390/ani12060771 (PMC8944629; doi:10.3390/ani12060771)
Supplement: Supplementary file 1 [file animals-12-00771-s001.zip › animals-1562176-supplementary.pdf]

Supplementary Table S1. Quantity of each antimicrobial that was used for dairy calves. Antimicrobial usage were expressed in used daily doses (UDD). The mean of the AMU for each antimicrobial was the simple arithmetic mean of the AMU for each antimicrobial (2.5%–97.5% quantile in brackets). There were 1 study in Canada, 2 studies in Ireland and 2 studies in Sweden.

**Country (Ref.)**		**Canada [1]**	**Ireland [2]**	**Ireland [3]**	**Sweden [4]**	**Sweden [4]**
Number of calves		310 (10 farms)	5358 (44 farms)	391 (14 farms)	27 (organic farms)	27 (conventiona l farms)
Purpose of use		diarrhea	treatment	overall use	treatment	treatment
β-lactam	Penicillins		2.8 (0–14)	0.15	0.58	1.17
	3rd & 4th generation cephalosporins		0.01 (0–0.54)			
	Amoxicillin/clavulanate			16.00		
	Ceftiofur	1.52		1.25		
Sulfonamides	Sulfonamides		18.4 (0–462)			
	Sulfamethazine	15.77 (0.62–37.50)				
	Trimethoprim/sulfonam ide	4.10 (0.35–9.93)		1.73	0.08	0.04
Aminoglycosides	Aminoglycosides		1.0 (0–9.3)			
	Dihydrostreptomycin				0.23	0.37
	Gentamycin			7.70		
	Spectinomycin			0.43		
	Framycetin or neomycin			3.01		
Phenicols	Phenicol		2.8 (0–42)			
	Florfenicol			14.13		
Tetracyclines	Tetracyclines		0.8 (0–9.3)	0.26		
	Oxytetracycline				0.04	
Quinolone	Fluoroquinolones		0.5 (0–7.3)	0.67		
	Enrofloxacin				0.01	0.01
Macrolides	Macrolides		1.7 (0–27.8)			

Supplementary Table S2. Antimicrobial resistance of bacteria associated with calf pneumonia. The number of resistant isolates divided by the total number of isolates and the corresponding 2.5%–97.5% quantile were provided in brackets. Study on *Pasteruella spp.* was in Spain and *Mannheimia spp.* in Belgium, respectively. Critically important antimicrobials (according to WHO) with the highest priority were in bold and italic.

**Class of Antimicrobial**	**Antimicrobial**	**Pasteurella spp.**		**Mannheimia spp.**	
		**Ref.**	**Proportion of Resistant Isolates**	**Ref.**	**Proportion of Resistant Isolates**
β-lactam	Amoxicillin/Clavu lanic acid	[5]	0.00 (0/37; 0.00–0.00)		
	Ampicillin	[5]	0.86 (32/37; 0.88–0.88)		
	Ceftiofur	[5]	0.00 (0/37; 0.00–0.00)		
	Cefoxitin	[5]	0.00 (0/37; 0.00–0.00)		
Sulfonamides	Sulfisoxazole	[5]	0.86 (32/37; 0.88–0.88)		

	Trimethoprim/Sulfamethoxazole	[5]	0.00 (0/37; 0.00–0.00)		
Aminoglycosides	Gentamicin			[6]	0.00(0/16; 0.00–0.00)
	Kanamycin	[5]	0.00 (0/37; 0.00–0.00)		
	Streptomycin	[5]	0.38 (14/37; 0.38–0.38)	[6]	0.94(15/16; 0.94–0.94)
Tetracyclines	Tetracycline	[5]	0.86 (32/37; 0.88–0.88)	[6]	0.94(15/16; 0.94–0.94)
Quinolone	***Ciprofloxacin***			[6]	0.25(4/16; 0.25–0.25)
Macrolides	***Erythromycin***			[6]	0.94(15/16; 0.94–0.94)

References

1. Gomez, D.E.; Arroyo, L.G.; Renaud, D.L.; Viel, L.; Weese, J.S. A multidisciplinary approach to reduce and refine antimicrobial drugs use for diarrhoea in dairy calves. *Vet. J.* **2021**, *274*, 105713, doi:10.1016/j.tvjl.2021.105713.
2. Earley, B.; Arguello, A.; O'Riordan, E.; Crosson, P.; Cappelleri, A.; McGee, M. Antimicrobial drug usage from birth to 180 days of age in Irish dairy calves and in suckler beef calves. *J. Appl. Anim. Res.* **2019**, *47*, 474–485, doi:10.1080/09712119.2019.1665525.
3. Gibbons, J.F.; Boland, F.; Buckley, J.F.; Butler, F.; Egan, J.; Fanning, S.; Markey, B.K.; Leonard, F.C. Patterns of antimicrobial resistance in pathogenic *Escherichia coli* isolates from cases of calf enteritis during the spring-calving season. *Vet. Microbiol.* **2014**, *170*, 73–80, doi:10.1016/j.vetmic.2014.01.010.
4. Sjöström, K.; Hickman, R.A.; Tepper, V.; Olmos Antillón, G.; Järhult, J.D.; Emanuelson, U.; Fall, N.; Sternberg Lewerin, S. Antimicrobial resistance patterns in organic and conventional dairy herds in Sweden. *Antibiot. (Basel, Switzerland)* **2020**, *9*,834, doi:10.3390/antibiotics9110834.
5. Maynou, G.; Bach, A.; Terré, M. Feeding of waste milk to Holstein calves affects antimicrobial resistance of *Escherichia coli* and *Pasteurella multocida* isolated from fecal and nasal swabs. *J. Dairy Sci.* **2017**, *100*, 2682–2694, doi:10.3168/jds.2016-11891.
6. Catry, B.; Decostere, A.; Schwarz, S.; Kehrenberg, C.; de Kruif, A.; Haesebrouck, F. Detection of tetracycline-resistant and susceptible *Pasteurellaceae* in the nasopharynx of loose group-housed calves. *Vet. Res. Commun.* **2006**, *30*, 707–715, doi:10.1007/s11259-006-3347-8.
